# Molecular docking and dynamics studies to identify novel active compounds targeting potential breast cancer receptor proteins from an indigenous herb *Euphorbia thymifolia* Linn

**DOI:** 10.12688/f1000research.146862.1

**Published:** 2024-04-25

**Authors:** Vasavi Kumblekar, Reshma Kumarchandra, K Sreedhara Ranganath Pai, ShamaPrasada K, Suman Manandhar, Rajeshwari Shastry, Sharada Rai

**Affiliations:** 1 Department of Biochemistry, Kasturba Medical College, Mangalore, Manipal Academy of Higher Education, Manipal, Karnataka, 576104, India; 2 Department of Pharmacology, Manipal College of Pharmaceutical Sciences, Manipal Acaedemy of Higher Education, Manipal, Karnataka, 576104, India; 3 Department of Cell and Molecular Biology, Manipal School of Life Science, Manipal Academy of Higher Education, Manipal, Karnataka, 576104, India; 4 Department of Pharmacology, Kasturba Medical College, Mangalore, Manipal Academy of Higher Education, Manipal, Karnataka, 576104, India; 5 Department of Pathology, Kasturba Medical College, Mangalore, Manipal Academy of Higher Education, Manipal, Karnataka, 576104, India

**Keywords:** Euphorbia thymifolia, phytoconstituents, breast cancer, anti-cancer activity, molecular docking, molecular dynamics, breast cancer receptor proteins, lead compounds.

## Abstract

**Background:**

Breast cancer has become the most prevalent disease and its incidence has almost doubled in the Indian population. This increased burden demands new targeted therapies with novel compounds either synthetically produced or derived from indigenous plants, which could be a promising approach for the development of drugs. *Euphorbia thymifolia* L is a widely growing tropical herb that has been reported to have various ethnopharmacological properties, including anticancer properties. Therefore, the aim of the present study was to screen the phytoconstituents and identify the active compounds present in the methanolic extract of *E. thymifolia* (ME.ET) as ligands to inhibit potential protein targets implicated in breast cancer using an *In-silico* approach.

**Methods:**

ME.ET was subjected to GC-MS analysis to screen the phytoconstituents, and the identified compounds were docked with protein targets such as extracellular signal-regulated kinases (ERK1), a serine/threonine kinase-1(AKT1), human epidermal growth factor 2 (HER2), estrogen receptor (ER), maternal embryonic leucine zipper kinase (MELK), polo-like kinase-1(PLK1), and protein tyrosine kinase (PTK6). Compounds with good docking scores were further subjected to dynamic studies to understand the protein ligand binding stability, ligand pathway calculation, and molecular mechanics energies combined with Poisson-Boltzmann (MM/PBSA) calculations using the Schrodinger suite.

**Results:**

GC-MS analysis revealed the presence of 245 phytoconstituents, 219 of which were unique. When subjected to docking, these phytocompounds, namely 3,6,9,12-tetraoxatetradecane-1,14-diyl dibenzoate (TTDB) and succinic acid, 2-(dimethylamino) ethyl 4-isopropylphenyl ester (SADPE), showed good docking scores. Molecular dynamics studies showed a high affinity and low binding energy for TTDB with HER2, ERK1, and SADPE with ER.

**Conclusions:**

Hence, in this study, we identified two lead compounds in *E.thymifolia* linn. Further *invitro* and *invivo* anticancer studies can be performed to confirm these results and to understand the molecular mechanism by which they exhibit anticancer activity against breast cancer.

## Introduction

Breast cancer is the most common and serious disease among women worldwide, affecting their health and socioeconomic status. The incidence of breast cancer has significantly increased and almost doubled compared to the previous decade, with up to 25.8 per 100,000 women among the Indian population. The incidence of breast cancer is higher in younger women and tends to be more aggressive owing to the molecular complexity of the tumor microenvironment ( Malvia et al. 2017). The treatment strategy for breast cancer depends on its subtype and is based on the presence or absence of estrogen (ER), progesterone (PR), and human epidermal growth factor 2 (HER 2) receptors ( Waks and Winer 2019). Tumors with hormone receptor-positive subtypes receive endocrine therapy or targeted antibody treatment; however, triple-negative breast cancer is one of the most aggressive types of breast cancer, with a high recurrence rate, making it the most difficult to treat ( Palma et al. 2015). Targeted therapy with anticancer agents in combination with immunotherapy is one of the treatment strategies for TNBC. However, there is a need for new and efficient compounds to target the proteins implicated in the tumorigenesis of TNBC ( Chang-Qing et al. 2020).

Targeting cell surface protein receptors can be an effective way to target malignant cells. Overexpression of the cell surface receptor HER2 proteins has been observed in patients diagnosed with invasive HER2 + breast cancer. It plays an important role in the growth and differentiation of epithelial cells ( Yousuf et al. 2017). HER2/EGFR triggers one of the major signalling pathways which involves PI3 kinase, Ras-Raf-MAPK pathway involved in cell survival, proliferation, migration and angiogenesis ( Balz et al. 2012). The estrogen receptor is another important target protein involved in the cross-talk between other signalling pathways and is implicated in the resistance to hormonal treatments in ER-positive breast cancer patients ( Zhang et al. 2020). Protein tyrosine kinase (PTK6) is another important protein target associated with ER+ BC. Increased PTK6 expression is associated with poor overall survival ( Ito et al. 2017). AKT1, a serine/threonine kinase, also known as protein kinase B and extracellular signal-regulated kinases (ERK1), plays a major role in cell proliferation, differentiation, migration, and anti-apoptotic effects and is overexpressed in TNBC ( Martorana et al. 2021; Gagliardi et al. 2020; Guo et al. 2020). Maternal embryonic leucine zipper kinase (MELK), another pivotal member of the serine/threonine kinase family, is a cell cycle regulator whose overexpression in BC is associated with poor prognosis ( Jiang and Zhang 2013). Therefore, these proteins were chosen as targets to screen and identify the most active compounds against both hormone receptor-positive BC and TNBC.

*Euphorbia thymifolia* known as laghudhika or choti-dudhi, is a herb traditionally used to treat blood dysentery, inflammation, asthma, and stomach pain ( Nadkarni and Nadkarni 2007). This herb has been reported to have antimicrobial, antidiabetic, antiarthritic, antiviral, and anti-inflammatory effects ( Mali and Panchal, 2013). However, the anticancer activity of this compound has not yet been reported so far ( Salehi et al. 2019). Thus, we have studied and reported the anticancer activity of the methanolic extract of *E. thymifolia* for the first time in our previous study.

Identification of the active compounds responsible for anticancer activity by traditional methods such as bioactivity-guided fractionation is a very elaborate, time-consuming, and laborious method. Recent advances in simulation technology have provided scope for basic in *In silico* studies, which are reliable and efficient for the identification of active compounds ( Kumari *et al*. 2014). Hence, we screened and analyzed all the phytocompounds identified in the GC-MS analysis of the methanolic extract of *E.thymifolia* using a structure-based virtual screening *in silico* approach to identify potential targets implicated in breast cancer ( Pinzi and Rastelli 2019; Herrera-Calderon et al. 2020).

### Objectives

Screening and identification of the active components of *Euphorbia thymifolia* and identifying their potential targets implicated in breast cancer through Insilico approach.

## Methods

### Study design

Plant material collection and extract preparation by cold maceration method, GCMS analysis, Insilico molecular docking and molecular dynamics study.

### Study setting - Plant material collection and extraction

The aerial parts of *Euphorbia thymifolia* L. were procured from the uncultivated areas of the Udupi District from April to June. The plant material was identified and authenticated by taxonomist Dr. K. Gopalakrishna Bhat, Professor of Botany (Rtd.) Poorna Prajna College, Udupi, The plant sample was deposited in the Herbarium of the Department of Pharmacognosy, Manipal College of Pharmaceutical Sciences. (Herbarium Voucher no: PP624) The aerial parts of *Euphorbia thymifolia* L. were collected, washed thoroughly and carefully with distilled water to remove all the soil and debris. The plant material was shade-dried at room temperature for 15 d until it was dry and crisp. After drying the shoots of *E. thymifolia* L., they were powdered and stored at 4°C.

### Methanolic extract by cold maceration method

The crude extract (10% w/v) 35 g in 350 ml was prepared by cold maceration as described by Abubakar et al using methanol procured from NICE Chemicals Private Limited (MI2037; B.no 103168) as the solvent for 72 h with intermittent shaking at room temperature. The extracts were then filtered through Whatman No. 1 filter paper. The clear extracts were concentrated using a rotary vacuum flash evaporator and freeze-dried by lyophilization, a process of drying the extract under controlled temperature and pressure using a lyophilizer. These lyophilized crude extracts were stored at 4°C until further use ( Abubakar and Haque 2020).

### GC-MS analysis

The composition of the methanolic extract of *E.thymifolia* was analyzed by GC-MS at Analytical Research & Metallurgical Laboratories Pvt. Ltd. Bangalore, India.

### In silico methodology: Selection of the lead compound by Molecular docking

Molecular docking was performed using Schrodinger suite to select the best protein-ligand complex. Commercial Maestro software version 11.8 (OPLS3e force field) was utilized for all the simulation studies. Protein preparation wizard was used for preparation of protein using the panel review, modify, and refine modules where the side chains and residues were filled, and water molecules beyond 3 Å were removed. GLIDE panel was used for receptor-grid generation to create a grid and locate the receptor at the binding site for the ligands to be docked. The size of the grid box generated at the inbound ligand site for each protein was 10 × 10 × 10 Å ( Rathi et al. 2019).

The crystal structures of the proteins were retrieved from the Protein Data Bank ( Berman et al. 2000). ERK1 (Protein Data Bank ID: 4QTB, Crystallographic precision of the PDB: 1.40 Å) bound to a piperazine-phenyl-pyrimidine derivative ( Chaikuad et al. 2014); AKT (Protein Data Bank ID: 4EKL, Crystallographic precision of the PDB: 2.00 Å) with an ATP site inhibitor which is a piperazine- pyrimidine derivative ligand ( Lin et al. 2012); EGFR/HER2 (Protein Data Bank ID: 1XKK, Crystallographic precision of the PDB: 2.40 Å) with bound ligand Lapatinib is a tyrosine kinase inhibitor in clinical development for cancer ( Wood et al. 2004); ER (Protein Data Bank ID: 2IOG, Crystallographic precision of the PDB: 1.60 Å) with indole ligand ( Dykstra et al. 2007); The crystal structure of MELK (PDB ID: 5K00, Crystallographic precision of the PDB: 1.77 Å) with a ligand which is an amide derivative ( Chen et al. 2017); Crystal structure of PLK1 (PDB ID: 4J52, Crystallographic precision of the PDB: 2.30 Å) with pyrimidodiazepinone as ligand ( Nie et al. 2013), and Crystal structure of PTK6 (PDB ID: 5H2U,Crystallographic precision of the PDB: 2.24 Å) with ligand Dasatinib ( Thakur et al. 2017).

The structures of all 219 phytocompounds were either retrieved from PubChem or derived using Chem-Draw software. These 219 ligands were imported into maestro and “ LIGPREP” panel was utilized for ligand preparation ( Rathi et al. 2019). Docking was performed using the Maestro Glide Module. Initially, all ligands were docked in standard precision (SP) mode, followed by extra precision (XP) mode to obtain a ligand docking XP score ( Elokely and Doerksen 2013).

### Free ligand binding energy calculation by Maestro (MM-GBSA)

All XP docking files corresponding to each phytocompound and the seven proteins were then subjected to MM-GBSA free ligand energy calculation using the PRIME module to understand the binding energy of the target protein listed above with all 219 phytocompounds. ( Ignjatović et al. 2016).

### Molecular dynamic (MD) simulations

To determine the protein ligand stability under the simulated physiological environment, the DESMOND panel of Maestro was used for the MD simulation assessment, which was executed on HER2 (1XKK), ERK1 (4QTB) with 3,6,9,12-tetraoxatetradecane-1,14-diyl dibenzoate (TTDB) (molecular formula: C24H30O8), and ER(2IOG) with succinic acid, 2-(dimethylamino) ethyl 4-isopropylphenyl ester (SADPE) (molecular formula: C17H25NO4) for 100 ns using the XP docking file. Three steps, namely the system builder, minimization, and MD simulation, were involved in this process. A predefined simple point charge model (SPC) was used to create an orthorhombic boundary for the XP docked complex of TTDB with 1XKK, 4QTB, and 2IOG with SADPE. The charges were neutralized, where three positive charges were neutralized by the addition of three chloride ions, and one negative charge was neutralized by one sodium ion. An isothermal–isobaric (NPT) ensemble with a constant temperature of 300 K and pressure of 1 bar was maintained throughout the simulation for 100ns. To determine the stability of the complex, the Root Mean Square Deviation (RMSD) was analyzed along with the protein–ligand contact timeline and covalent/non-covalent interactions ( Ivanova et al. 2018).

## Results

### GCMS analysis

GC-MS analysis of the methanolic extract of *E.thymifolia* showed 23 prominent peaks, with each of the retention time peaks having several hits comprising a total of 245 hits, as shown in Supplemental Data File 1. Each of these hits was recognized by comparing their retention time peak, peak area (%), and height to those of the already known compounds identified by the National Institute of Standards and Technology (NIST) library. Of the 245 phytocompounds, duplicates were removed and 219 unique compounds were identified in the methanolic extract of *E.thymifolia.* The identified phytocompounds were organic acids, phenyl esters, hydrocarbons, saturated and unsaturated cyclic compounds, and fatty acids.

### Molecular docking score and binding energy

The results of XP molecular docking using GLIDE were as follows: All 219 ligands with each of the seven proteins were comprehensively analyzed with their docking scores and amino acid interactions. The ligands that showed good interaction with most of the proteins were 3,6,9,12-tetraoxatetradecane-1,14-diyl dibenzoate (TTDB), succinic acid, and 2-(dimethylamino) ethyl 4-isopropylphenyl ester (SADPE), as shown in Table 1. TTDB showed very good interaction with two proteins, namely 1XKK and 4QTB, with docking scores: MMGBSA ΔG binding energies of -8.627, -65.22 kcal/mol and -10.112 and -71.103 kcal/mol with two types of non–covalent interactions, respectively. Similarly, 2IOG with SADPE exhibited a docking score of -8.865 and MMGBSA ΔG binding energy of -45.94 kcal/mol with two types of non-covalent interactions.

**Table 1.** Summary of the Docking results of the ligands chosen.

Name and structure of the ligand	Target proteins with PDB ID	XP GScore	MMGBSA ΔG Binding energies in kCal/mol
1. 3,6,9,12-tetraoxatetradecane-1,14-diyl dibenzoate (TTDB)	ERK 1 (4QTB)	-10.112	-71.103
	EGFR/HER2 (1XKK)	-8.6272	-65.22
2. Succinic acid, 2-(dimethylamino) ethyl 4-isopropylphenyl ester (SADPE)	ER (2IOG)	-8.84	-45.94

### Protein-ligand interactions

The amino acid residue Lys745 of the 1XKK protein showed a strong hydrogen bond and Phe856 showed pi-pi stacking with TTDB. Lys 71 and Met 125 of the protein 4QTB interacted with the oxy- group of TTDB. The amino acid residue Lys531 of 2IOG showed a hydrogen bond, and Trp383 showed pi-cation interactions with the ligand SADPE, as illustrated in Table 2. Hydrogen bond formation between a ligand and a protein plays a crucial role in drug design. Furthermore, the XP file obtained was subjected to the Prime MM-GBSA method to evaluate the binding affinity more accurately. The calculated binding energies (MM-GBSA ΔG) for 1XKK and 4QTB with the TTDB complex were − -65.228 kcal/mol and -71.103 kcal/mol respectively, and that of 2IOG with SADPE was -45.945 kcal/mol.

**Table 2.** Protein-ligand interaction diagram.

Protein-ligand interaction diagram	Amino acid residues involved in the interaction
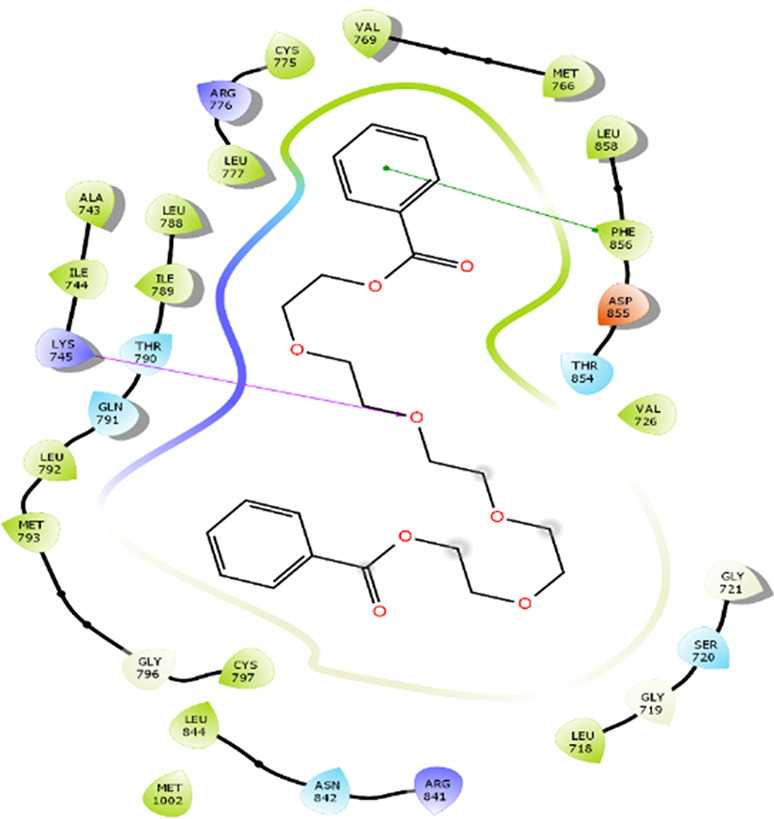	**1XKK with TTDB:** Hydrogen bond with Lys745 Pi-Pi stacking with Phe856
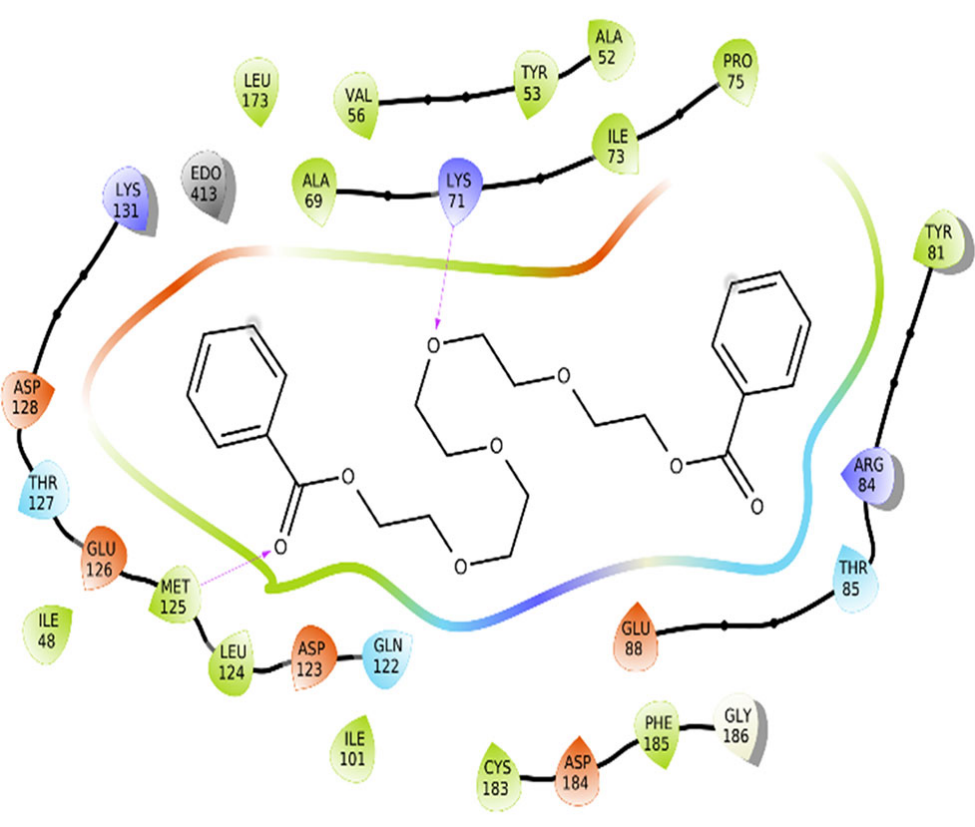	**4QTB with TTDB:** Hydrogen bonds with Lys71 and Met125
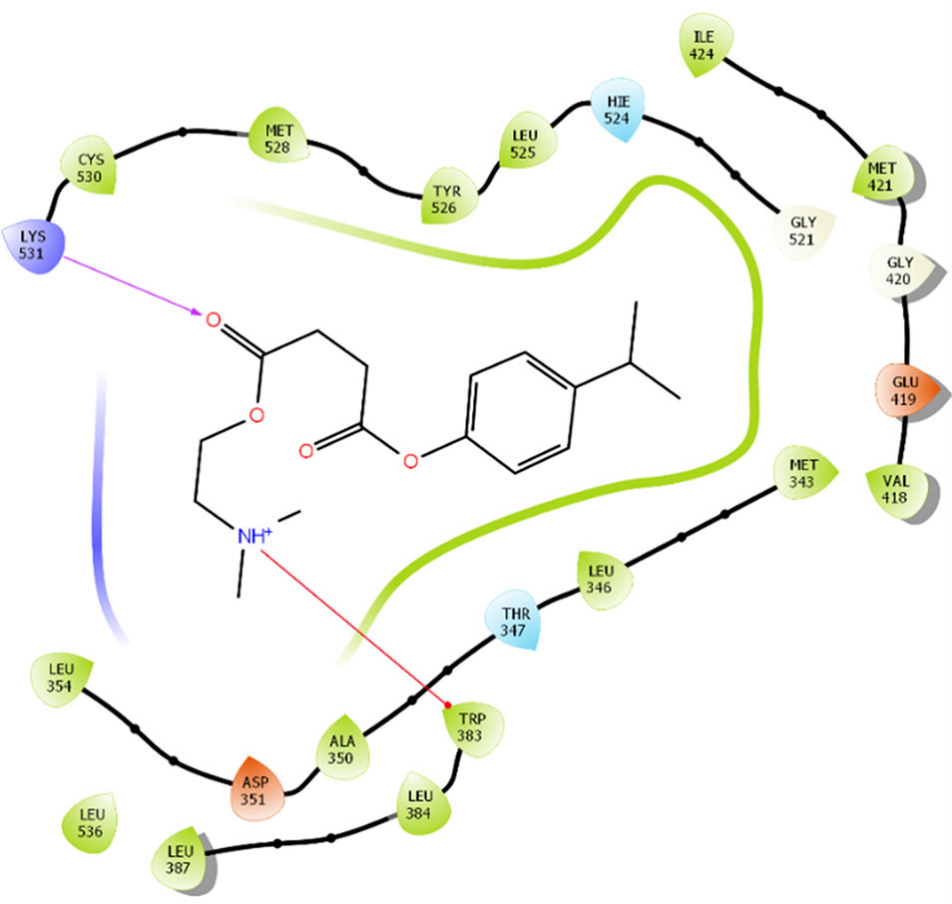	**2IOG with SADPE:** Hydrogen bond with Lys531 Pi-Cation with Trp383

### Molecular dynamics

Furthermore, to understand the interaction between the protein and ligand complex, a molecular dynamics study was performed for 100 ns. The RMSD values obtained were explained to obtain more clarity regarding the binding stability of the docked complexes.

**Figure 1.**
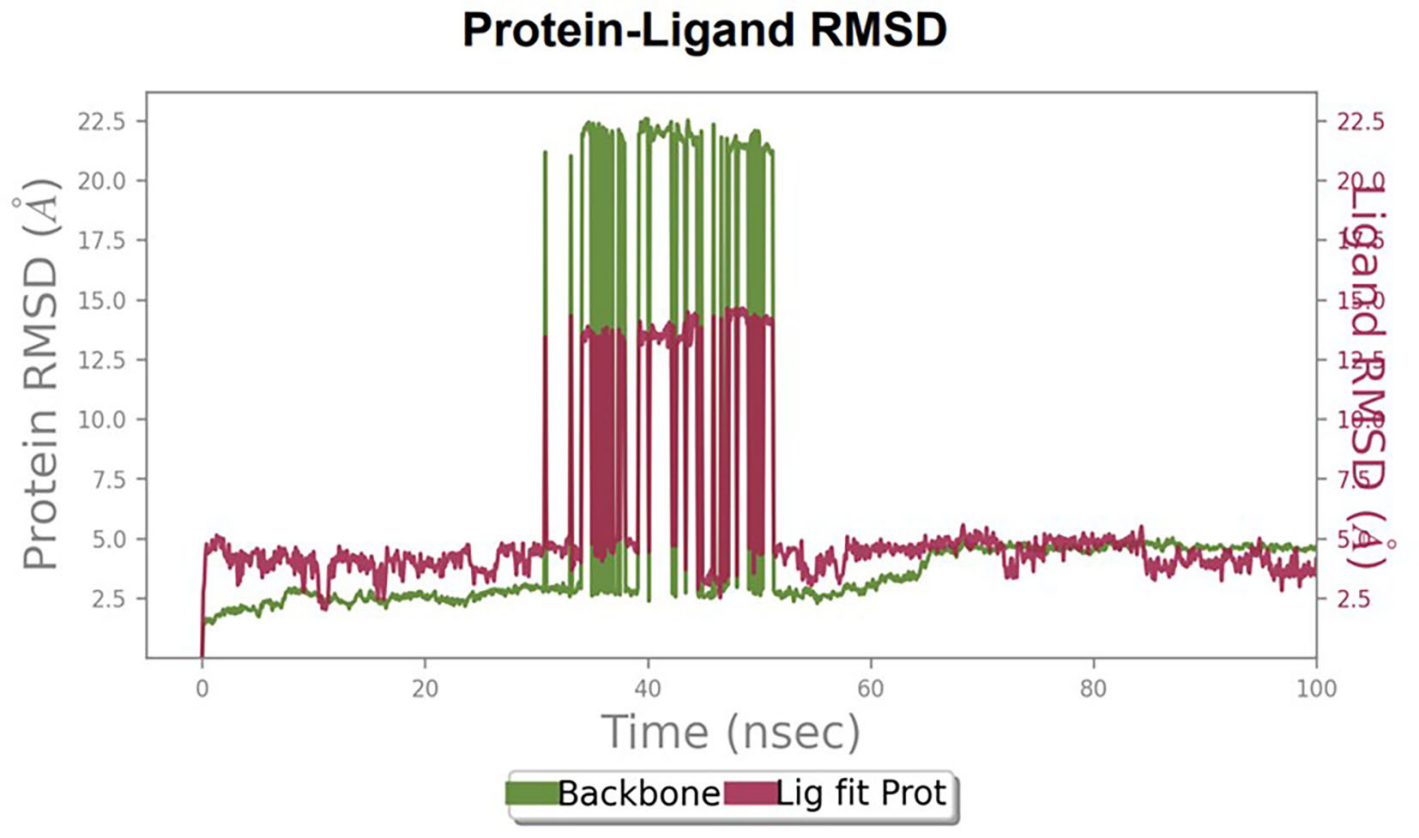
Protein-ligand root mean square deviation (RMSD) plot. RMSD plot of TTDB bound to active inhibitor site of HER2 (1XKK).

**Figure 2.**
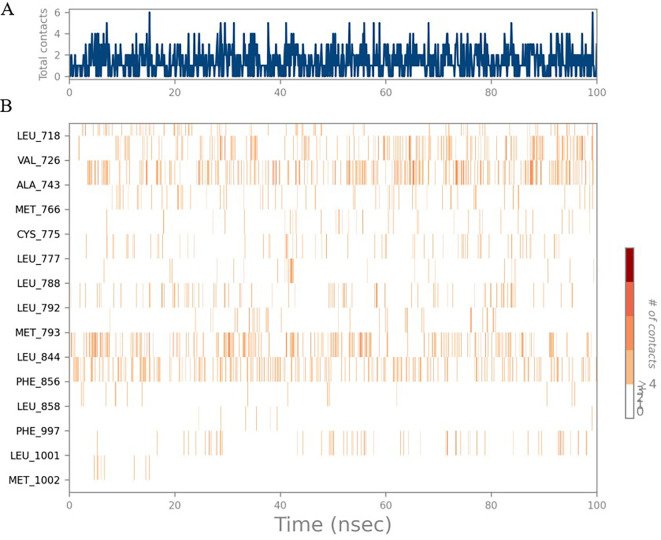
(A) Protein-ligand contact timeline plot. (B) Total contact timeline plot of TTDB bound to active inhibitory site at HER2 protein (IXKK).

**Figure 3.**
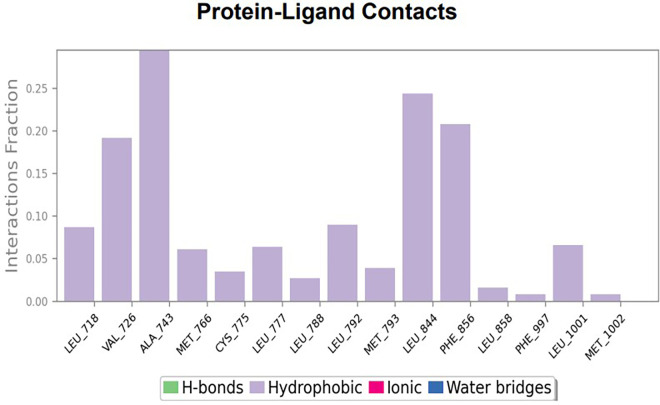
Bar diagram showing various types of interactions established at the amino acid residual sites during HER-2 protein-TTDB ligand simulation.

The RMSD values of HER2 (1XKK) and TTDB were found to be around 4.8 Å, 4.0 Å respectively. The RMSD plot of both ligand and protein was stable after 20 ns, as shown in Figure 1. Later, the protein-ligand complex showed varied fluctuations until 50ns and then remained stable for the next 50ns. The molecular dynamics trajectory showed that amino acid residues such as Leu718, Ala 743, and Leu844 were vital before ligand (TTDB) stabilization up to 20 ns. Amino acid residues such as Val726, Leu844, and Phe856 established contacts with the ligand to be crucial hotspots for equilibrium, as shown in Figures 2B and 3.

**Figure 4.**
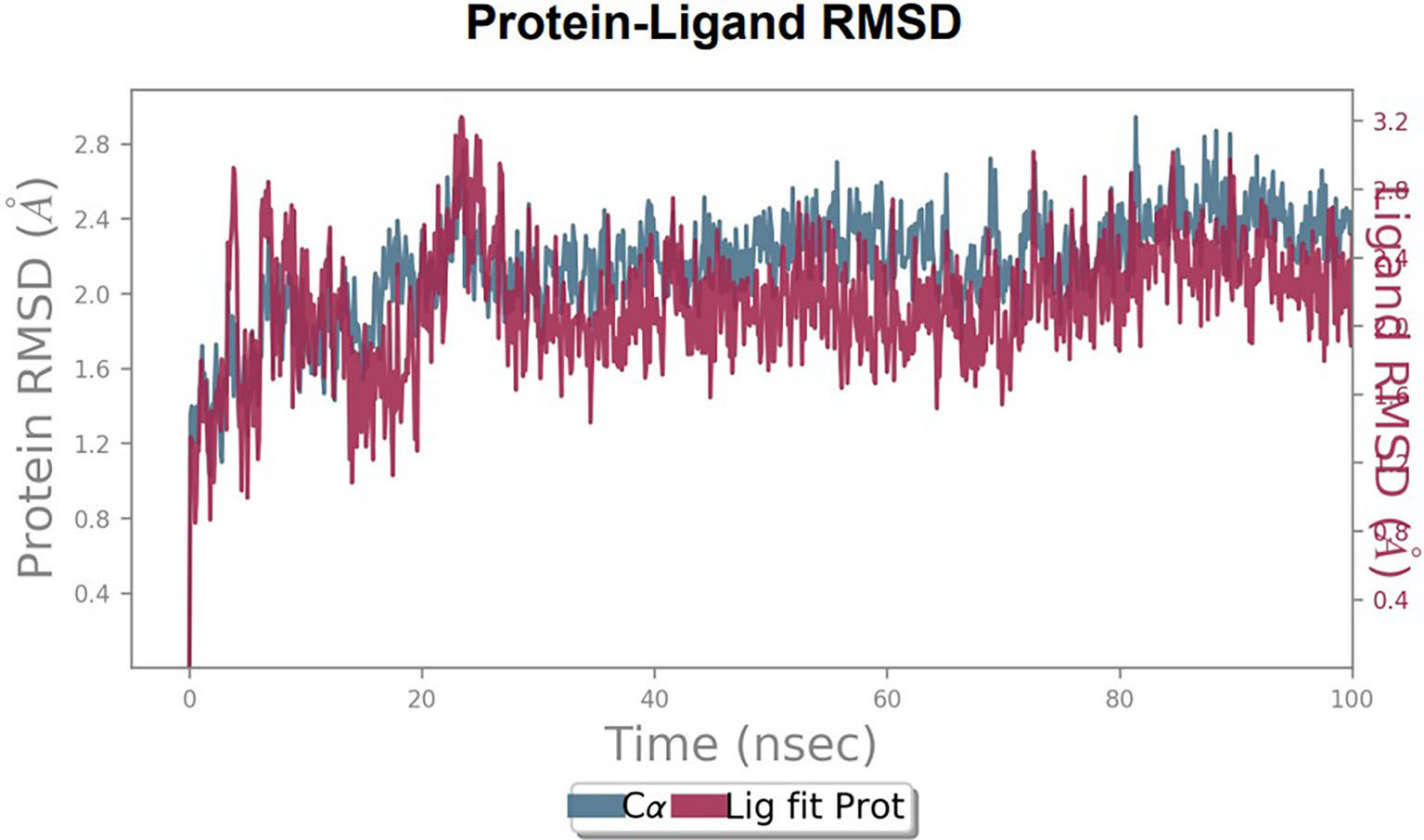
Protein-ligand root mean square deviation (RMSD) plot. RMSD plot of TTDB bound to active inhibitor site of ERK (4QTB).

**Figure 5.**
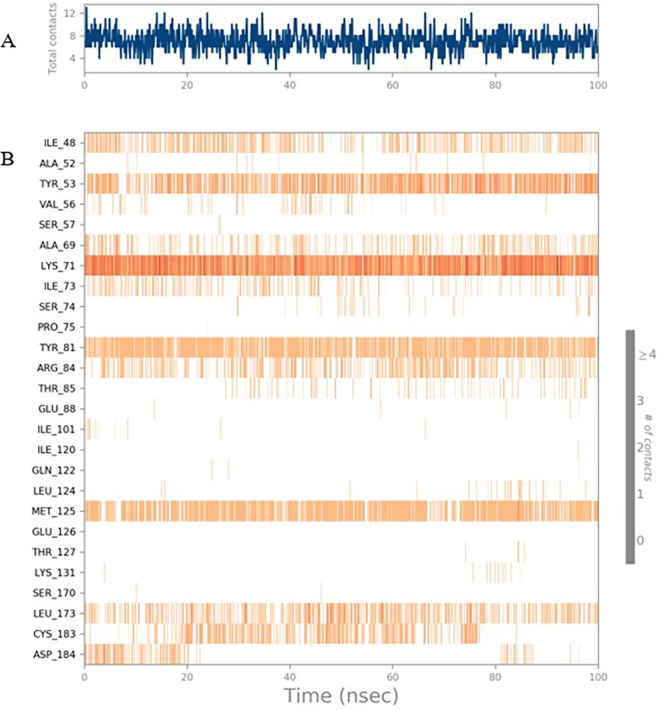
(A) Protein-ligand contact timeline plot. (B) Total contact timeline plot of TTDB bound to active inhibitory site at ERK 1 protein (4QTB).

**Figure 6.**
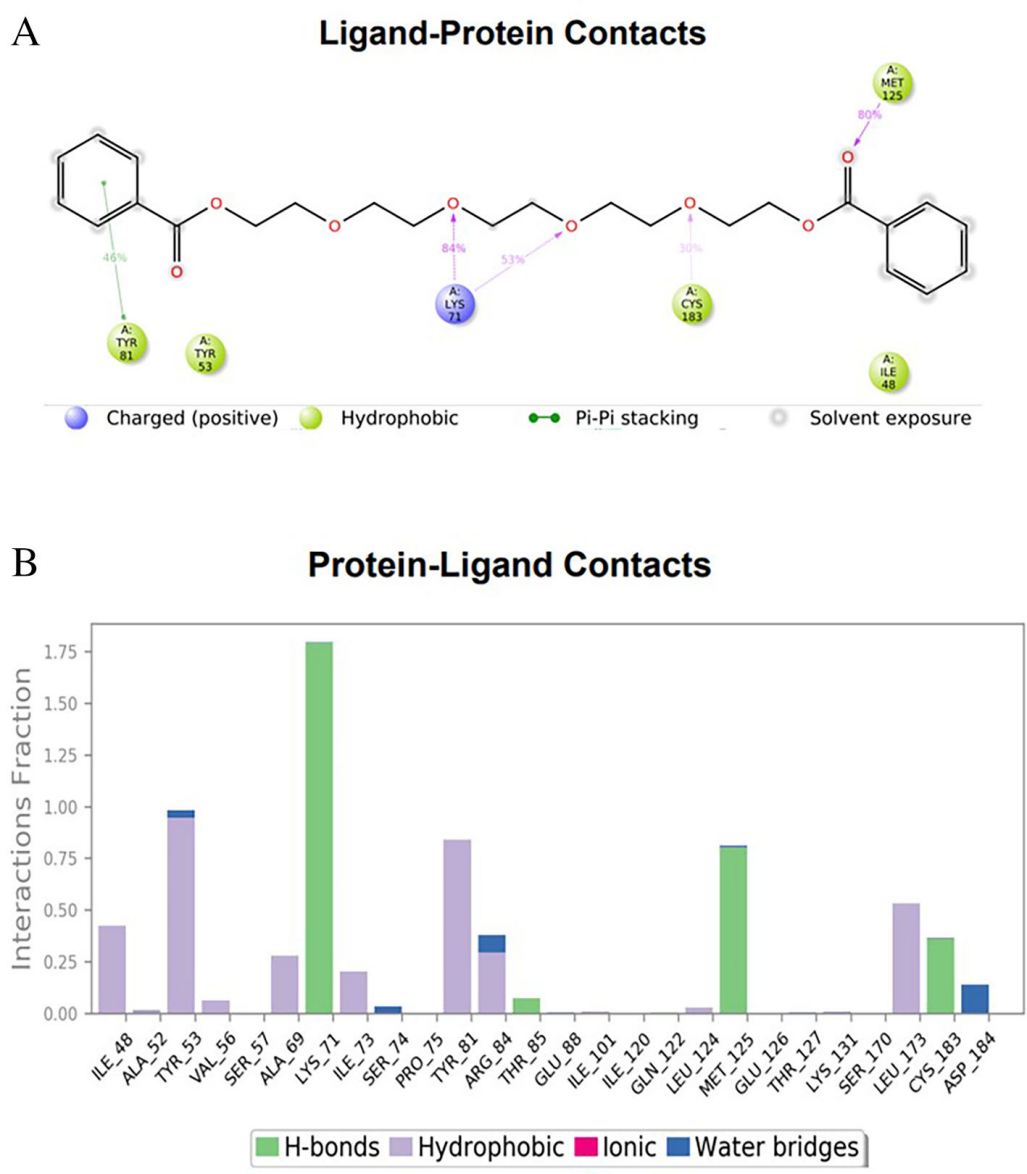
(A) Protein interaction diagram. (B) Bar diagram showing various types of interactions established at the amino acid residual sites during ERK-1 protein-TTDB ligand simulation.

To observe the protein-ligand interaction stability of TTDB with 4QTB, the RMSD values of the 4QTB with TTDB was found to be 2.2 Å and 2.4 Å respectively ( Figure 4). This complex exhibited the highest stability throughout the simulation without larger deviations. Amino acid residues, such as Ile 48, Ala 69, Ile73, Arg 84, and Asp 184, were significant in stabilizing the complex until 20 ns. Other amino acid residues, such as Tyr 53, Lys 71, Tyr 81, Met 125, Leu 173, Val 56, and Cys 183, showed strong contact with TTDB and contributed to their strong stabilization for 100 ns. TTDB showed a prominent interaction with ERK (4QTB) ( Figures 5B and 6 A & B).

**Figure 7.**
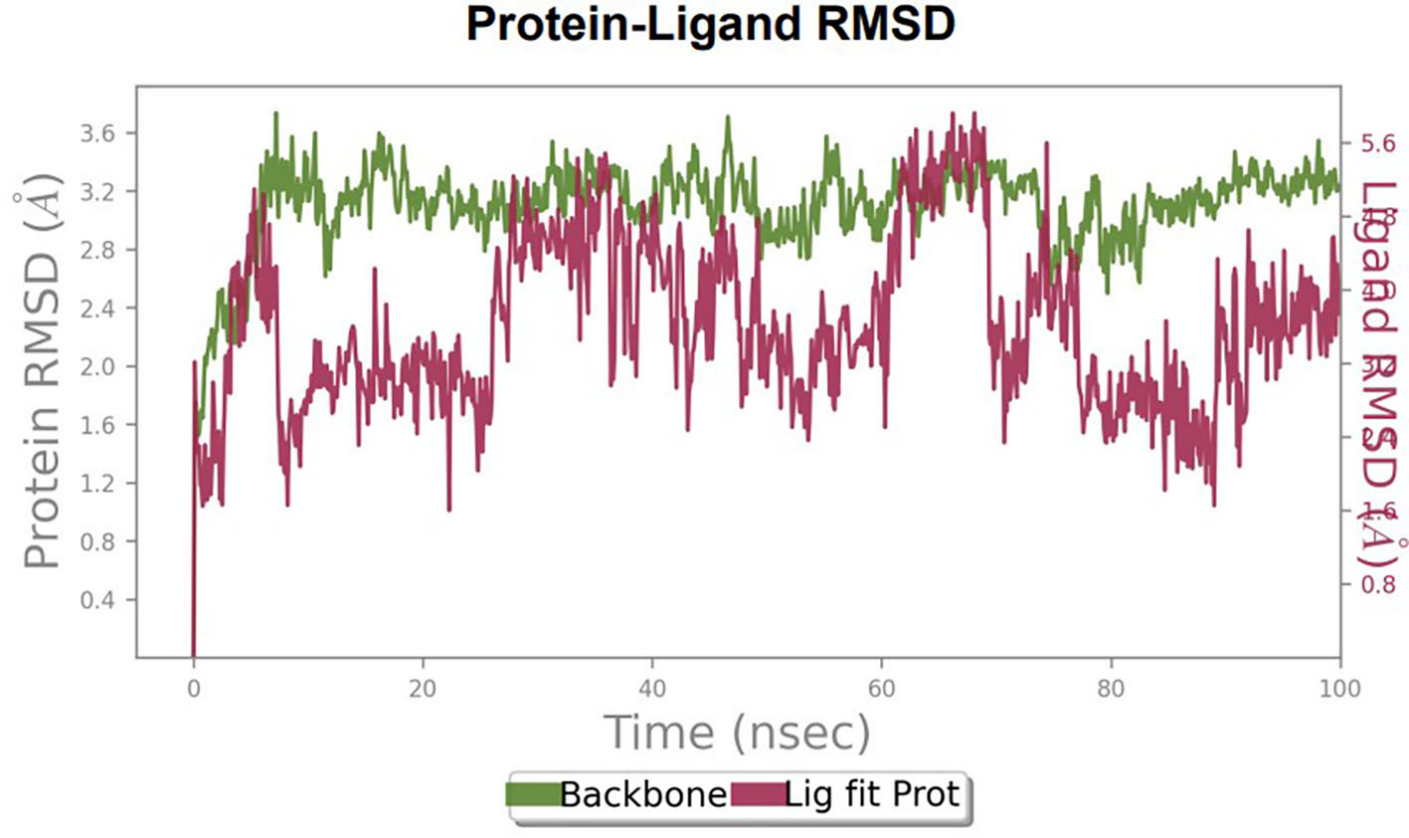
Protein-ligand root mean square deviation (RMSD) plot. RMSD plot of SADPE bound to active inhibitor site of ER protein (2IOG).

**Figure 8.**
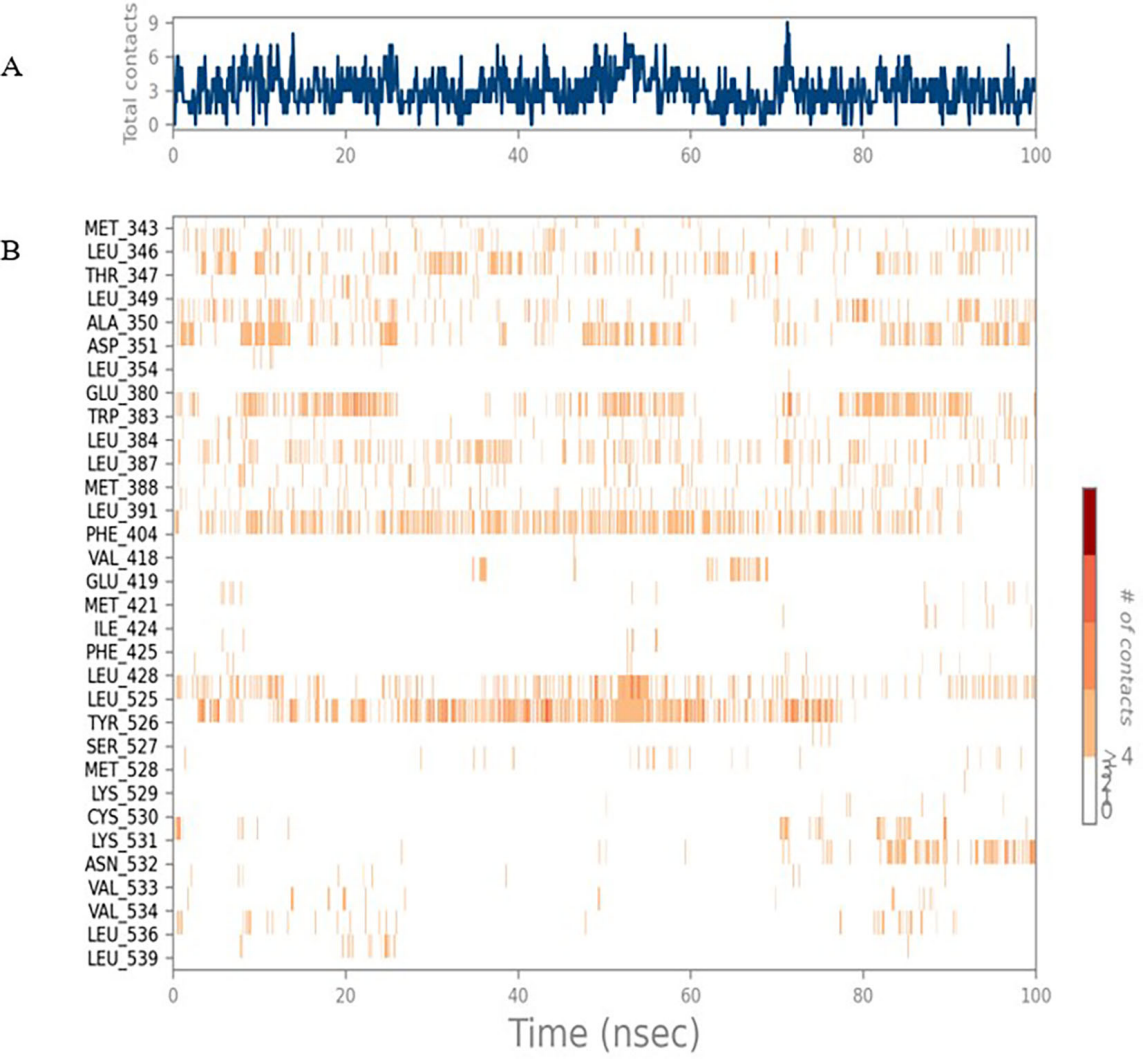
(A) Protein-ligand contact timeline plot. (B) Total contact timeline plot of SADPE bound to active inhibitory site at ER protein (21OG).

**Figure 9.**
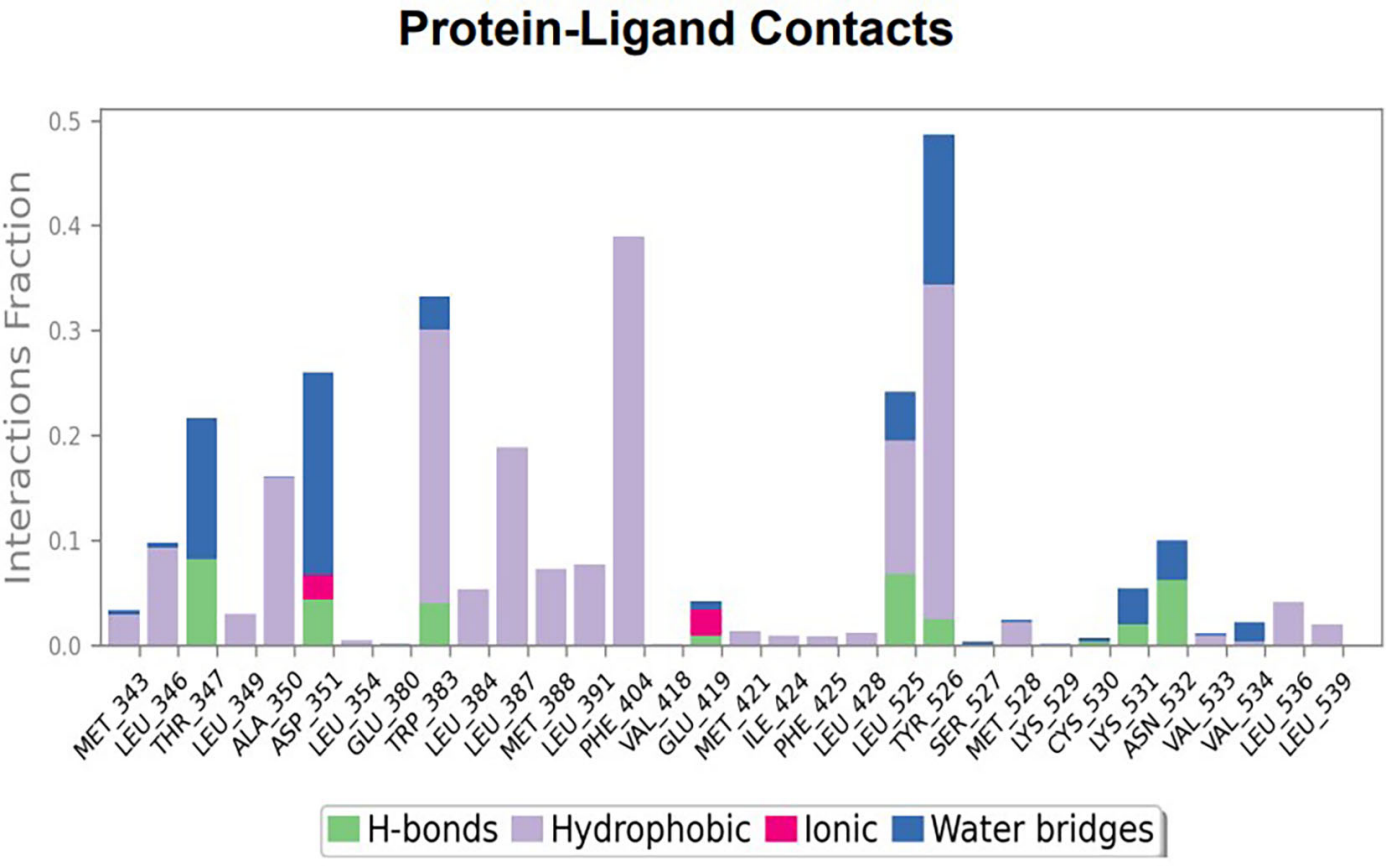
Bar diagram showing various types of interactions established at the amino acid residual sites during ER protein-SADPE ligand simulation.

The RMSD values of ER (2IOG) and SADPE were approximately 5.0 Å, 4.0 Å respectively. The RMSD plot of both ligands and proteins stabilized after 20 ns for some time ( Figure 7). Up to 20 ns, amino acid residues such as Thr347, Ala350, Leu 384, Asn532, Cys530, and Lys531 were important prior to ligand stabilization. Subsequently, the amino acid residues Asp351, Leu 387, Phe 404, Trp383, Leu525, and Tyr 526 showed strong interactions with the ligand, which contributed to stabilization ( Figures 8B and 9).

## Discussion

The phytocompounds were shortlisted based on their docking score, their interaction with more than one protein, and amino acid interactions. A literature search was carried out to investigate whether any of these compounds had anticancer activity. Chloride-substituted phenoxy acetic acid was used for the synthesis of a benzoyl hydrazide derivative, which has been reported to have *in vitro* antimicrobial activity ( Al-Ostoot et al. 2019). To date, no other studies have been reported. TTDB and SADPE showed the highest docking scores against the proteins implicated in the pathogenesis of breast cancer, such as HER2 (IXKK), ERK1 (4QTB), and ER (2IOG). The XP docking results provided insight into the establishment of a strong interaction between a ligand and a protein that contains important amino acid residues. The interaction diagram for all proteins revealed the formation of more than one non-covalent bond, which contributed to the stability of the complex, as described in Table 2. The binding affinity calculated for the protein–ligand complex supports this statement. From previous literature, RMSD values below 4 Å are considered good, which implies minimal protein-ligand fluctuations ( Hevener et al. 2009; Rao et al. 2021). In the simulations conducted for 100 ns for TTDB- HER2 & SADPE- ER, stability was established after 20 ns with non-covalent bond interactions. For the entire simulation, HER2 (IXKK) -TTDB had an average of four contacts with a maximum 6 and a minimum of two contacts ( Figure 2A), and the ER (2IOG)- SADPE complex showed an average of six contacts, with a maximum of 9 and a minimum of three contacts ( Figure 8A). The ERK1 (4QTB-TTDB) complex showed an average of eight contacts, with a maximum of 12 and a minimum of four contacts, with the highest stability number of interactions when compared to the other two complexes throughout the simulation ( Figure 5A). In the HER2 (IXKK-TTDB) complex, the majority of contacts were made only by hydrophobic interactions ( Figure 3). In TTDB- ERK1 complex Lys71 & Met 125 has made contact with the oxy groups for more than 80 % of the time. Other amino acids Tyr81 made a hydrophobic contact with the aromatic ring for approximately 46 % of the time and Cys 183 had hydrogen bonding with oxy group for 30 % of the time, as shown in the ligand-protein contact diagram ( Figure 6A). In the ER (2IOG-SADPE) complex, Tyr 526 formed a non-covalent interaction due to hydrogen bonding and hydrophobic interactions for most of the contact time ( Figure 9). In the case of HER2 and ERK1 protein complexes with TTDB, the activity is mainly attributed to the presence of an aromatic ring and multiple Oxy groups present in the ligand. The SADPE activity with the ER protein can be attributed to the presence of oxy groups. From the dynamics study, the ligand TTDB showed a profound interaction with ERK and was highly stable compared to its interaction with HER2. The SADPE-ER complex did not show a significant or stable interaction. Therefore, in our study, we report that TTDB has a greater affinity for binding to the ERK1 protein due to stable interactions, and therefore inhibits the protein. These *in silico* results can be further validated using an in *in-vitro* cytotoxicity assay. Hence, for the first time, we report that the anticancer activity of *E. thymifolia* L. could be attributed to the presence of the active compounds TTDB and SADPE in the crude methanolic extract.

## Conclusion

Considering the docking score, H bond, binding stability, and contact time between ligand and protein, TTDB and SADPE could be suggested as the active components of the methanolic extract of *E. thymifolia.* Furthermore, these two compounds can be synthesized and tested for their *in vitro* anticancer activity in breast cancer cell lines to confirm the *in silico* results.

## Data Availability

Dryad: “Molecular docking and dynamics studies to identify novel active compounds targeting potential breast cancer receptor proteins from an indigenous herb Euphorbia thymifolia Linn” DOI: 10.5061/dryad.rn8pk0pjt ( Kumblekar et al. 2024). This work was licensed under a CC0 1.0 Universal (CC0 1.0) Public Domain Dedication license. Dryad: STROBE checklist for ‘Molecular docking and dynamics studies to identify novel active compounds targeting potential breast cancer receptor proteins from an indigenous herb Euphorbia thymifolia Linn’ https://doi.org/10.5281/zenodo.10906175.

## Software availability statement

Molecular docking as mentioned in the study can also be performed using AutoDock a freely accessible alternative software.

### Extended data

Dryad: Supplementary data file 1 GCMS dataset: https://doi.org/10.5061/dryad.rn8pk0pjt ( Kumblekar et al. 2024).

This work is licensed under a CC0 1.0 Universal (CC0 1.0) Public Domain Dedication license.
