# Supplementary material for: Molecular docking and dynamics studies to identify novel active compounds targeting potential breast cancer receptor proteins from an indigenous herb Euphorbia thymifolia Linn
Source: F1000Res. 2024 Dec 9;13:385. Originally published 2024 Apr 25. [Version 3] doi: 10.12688/f1000research.146862.3 (PMC12199905; doi:10.12688/f1000research.146862.3)
Supplement: Peer review history

# Reviewer report: Reviewer response for version 3

**DOI:** 10.5256/f1000research.175619.r363588

**Published:** 2025-02-11

**Authors:** Subhash Chandra

**Affiliations:** 1 Soban Singh Jeena University, Kumaun University, Nainital, Uttarakhand, India

Review  report for the manuscript entitled “Molecular docking and dynamics studies to identify novel active compounds targeting potential breast cancer receptor proteins from an indigenous herb *Euphorbia thymifolia* Linn”

 Authors have done experimental work  to  show anti-cancer potential  of *Euphorbia thymifolia* Linn. In this regard,  they have conducted  GC-MS analysis to screen the phyto-constituents  and reported  245  phytochemicals. Finally, they conducted in silico work with many molecular  targets of cancer  to   reveal anti-cancer potential of this herb.

 Overall, it is a well-written manuscript.

 The title of this manuscript looks good.

 The introduction has been well described.

 Methods are written explaining every step. 

 The results  and  discussion are well   described .

 The conclusion sections explain very well the outcomes of this manuscript.

Is the work clearly and accurately presented and does it cite the current literature?

Yes

If applicable, is the statistical analysis and its interpretation appropriate?

Not applicable

Are all the source data underlying the results available to ensure full reproducibility?

Partly

Is the study design appropriate and is the work technically sound?

Yes

Are the conclusions drawn adequately supported by the results?

Yes

Are sufficient details of methods and analysis provided to allow replication by others?

Yes

Reviewer Expertise:

Drug discovery.

I confirm that I have read this submission and believe that I have an appropriate level of expertise to confirm that it is of an acceptable scientific standard.

# Reviewer report: Reviewer response for version 3

**DOI:** 10.5256/f1000research.175619.r346843

**Published:** 2024-12-09

**Authors:** Renukaradhya Math

**Affiliations:** 1 Shri Dharmasthala Manjunatheshwara University, Dharwad, India

Authors have made required changes in the manuscript and are satisfactory.

Is the work clearly and accurately presented and does it cite the current literature?

Partly

If applicable, is the statistical analysis and its interpretation appropriate?

Not applicable

Are all the source data underlying the results available to ensure full reproducibility?

Yes

Is the study design appropriate and is the work technically sound?

Yes

Are the conclusions drawn adequately supported by the results?

Yes

Are sufficient details of methods and analysis provided to allow replication by others?

Yes

Reviewer Expertise:

Microbiology, biotechnology and bioinformatics

I confirm that I have read this submission and believe that I have an appropriate level of expertise to confirm that it is of an acceptable scientific standard.

# Reviewer report: Reviewer response for version 2

**DOI:** 10.5256/f1000research.174634.r340321

**Published:** 2024-12-02

**Authors:** Renukaradhya Math

**Affiliations:** 1 Shri Dharmasthala Manjunatheshwara University, Dharwad, India

My first review comments:

 Comment 1. Abstract (Background): author states “ *Euphorbia thymifolia *L is a widely growing tropical herb that has been reported to have various ethnopharmacological properties, including anticancer properties but further in Introduction section they state “However, the anticancer activity of this compound has not yet been reported so far” two statements are contradictory. In fact a recent study by Sulaiman C.T. et al 2023 (Ref 1) profiled anti-cancer activity of two Euphorbia species (thymifolia and hirta).

 Author Response: We actually meant to state that Euphorbia genus in general has been reported to have various ethnopharmacological properties including anticancer properties. But the species of interest in our study namely *Euphorbia thymifolia* although was reported to have several ethnopharmacological properties, anticancer property was not reported as per the literature available, when we initiated the project (2019). However we can rectify the statement already mentioned in the manuscript if you permit us to do so.

 My second review Comment 1: authors are free to rectify their statements in the manuscript, if required/necessary.

 Comment 2: Introduction section: author state “we have studied and reported the anticancer activity of the methanolic extract of *E. thymifolia *for the first time in our previous study” reference is missing!

 Author Response: We have studied the invitro anticancer activity of the methanolic extract of *E. thymifolia. *This original article has been communicated to the journal "Jordan Journal of Pharmaceutical Sciences" for possible publication, Hence the reference could not be cited until published.

 My second review comment 2: authors can cite their previous work in the manuscript as unpublished along with details of title, author and submitted journal.

Is the work clearly and accurately presented and does it cite the current literature?

Partly

If applicable, is the statistical analysis and its interpretation appropriate?

Not applicable

Are all the source data underlying the results available to ensure full reproducibility?

Yes

Is the study design appropriate and is the work technically sound?

Yes

Are the conclusions drawn adequately supported by the results?

Yes

Are sufficient details of methods and analysis provided to allow replication by others?

Yes

Reviewer Expertise:

Microbiology, biotechnology and bioinformatics

I confirm that I have read this submission and believe that I have an appropriate level of expertise to confirm that it is of an acceptable scientific standard, however I have significant reservations, as outlined above.

# Response

**Published:** 2024-12-04

**Authors:** Vasavi Kumblekar

**Affiliations:** Biochemistry, Manipal Academy of Higher Education, Mangalore, Karnataka, India

Answer to Comment 2: 

 Dear Sir, 

 As suggested, reference for the unpublished work has been cited in the manuscript and added in the bibliography.

# Reviewer report: Reviewer response for version 1

**DOI:** 10.5256/f1000research.160986.r324470

**Published:** 2024-10-09

**Authors:** Renukaradhya Math

**Affiliations:** 1 Shri Dharmasthala Manjunatheshwara University, Dharwad, India

Manuscript entitled “Molecular docking and dynamics studies to identify novel active compounds targeting potential breast cancer receptor proteins from an indigenous herb *Euphorbia thymifolia* Linn” by Kumblekar et al. is written well. My comments are below,

**1. Abstract (Background)**: author states “ *Euphorbia thymifolia *L is a widely growing tropical herb that has been reported to have various ethnopharmacological properties, including anticancer properties but further in Introduction section they state “However, the anticancer activity of this compound has not yet been reported so far” two statements are contradictory. In fact a recent study by Sulaiman C.T. et al 2023 (Ref 1) profiled anti-cancer activity of two Euphorbia species (thymifolia and hirta).

 2. **Introduction section**: author state “we have studied and reported the anticancer activity of the methanolic extract of *E. thymifolia *for the first time in our previous study” reference is missing!

Is the work clearly and accurately presented and does it cite the current literature?

Partly

If applicable, is the statistical analysis and its interpretation appropriate?

Not applicable

Are all the source data underlying the results available to ensure full reproducibility?

Yes

Is the study design appropriate and is the work technically sound?

Yes

Are the conclusions drawn adequately supported by the results?

Yes

Are sufficient details of methods and analysis provided to allow replication by others?

Yes

Reviewer Expertise:

Microbiology, biotechnology and bioinformatics

I confirm that I have read this submission and believe that I have an appropriate level of expertise to confirm that it is of an acceptable scientific standard, however I have significant reservations, as outlined above.

## References

[1] : Metabolite profiling and anti-cancer activity of two medicinally important Euphorbia species. *Medicine in Omics* .2023;7: 10.1016/j.meomic.2022.100018 10.1016/j.meomic.2022.100018

# Response

**Published:** 2024-10-09

**Authors:** Vasavi Kumblekar

**Affiliations:** Biochemistry, Manipal Academy of Higher Education, Mangalore, Karnataka, India

Dear Dr. Renukaradhya Math, 

 Thank you for taking your precious time to review our article and for the critical analysis. We would like to give our clarifications for the queries raised. 

 Comment 1. Abstract (Background): author states “ *Euphorbia thymifolia *L is a widely growing tropical herb that has been reported to have various ethnopharmacological properties, including anticancer properties but further in Introduction section they state “However, the anticancer activity of this compound has not yet been reported so far” two statements are contradictory. In fact a recent study by Sulaiman C.T. et al 2023 (Ref 1) profiled anti-cancer activity of two Euphorbia species (thymifolia and hirta).

 Response: We actually meant to state that Euphorbia genus in general has been reported to have various ethnopharmacological properties including anticancer properties. But the species of interest in our study namely *Euphorbia thymifolia* although was reported to have several ethnopharmacological properties, anticancer property was not reported as per the literature available, when we initiated the project (2019). However we can rectify the statement already mentioned in the manuscript if you permit us to do so.

 Comment 2: Introduction section: author state “we have studied and reported the anticancer activity of the methanolic extract of *E. thymifolia *for the first time in our previous study” reference is missing!

 Response: We have studied the invitro anticancer activity of the methanolic extract of *E. thymifolia. *This original article has been communicated to the journal "Jordan Journal of Pharmaceutical Sciences" for possible publication, Hence the reference could not be cited until published.

 Requesting you to kindly review the responses and do the needful.

 Thanking You,

 Yours Sincerely

 Vasavi K
